# Cytokines in Male Fertility and Reproductive Pathologies: Immunoregulation and Beyond

**DOI:** 10.3389/fendo.2017.00307

**Published:** 2017-11-20

**Authors:** Kate L. Loveland, Britta Klein, Dana Pueschl, Sivanjah Indumathy, Martin Bergmann, Bruce E. Loveland, Mark P. Hedger, Hans-Christian Schuppe

**Affiliations:** ^1^Centre for Reproductive Health, Hudson Institute of Medical Research, Clayton, VIC, Australia; ^2^Department of Molecular and Translational Sciences, School of Clinical Sciences, Monash Medical Centre, Monash University, Clayton, VIC, Australia; ^3^Department of Anatomy and Developmental Biology, Monash University, Clayton, VIC, Australia; ^4^Institute of Veterinary Anatomy, Histology and Embryology, Justus Liebig University Giessen, Giessen, Germany; ^5^Institute of Anatomy and Cell Biology, Justus Liebig University Giessen, Giessen, Germany; ^6^Burnet Institute, Melbourne, VIC, Australia; ^7^Department of Urology, Pediatric Urology and Andrology, Justus Liebig University Giessen, Giessen, Germany

**Keywords:** cytokines, testis cancer, immune cells, macrophages, spermatogenesis, infertility, spermatogonial stem cells, male reproductive health

## Abstract

Germline development *in vivo* is dependent on the environment formed by somatic cells and the differentiation cues they provide; hence, the impact of local factors is highly relevant to the production of sperm. Knowledge of how somatic and germline cells interact is central to achieving biomedical goals relating to restoring, preserving or restricting fertility in humans. This review discusses the growing understanding of how cytokines contribute to testicular function and maintenance of male reproductive health, and to the pathologies associated with their abnormal activity in this organ. Here we consider both cytokines that signal through JAKs and are regulated by SOCS, and those utilizing other pathways, such as the MAP kinases and SMADs. The importance of cytokines in the establishment and maintenance of the testis as an immune-privilege site are described. Current research relating to the involvement of immune cells in testis development and disease is highlighted. This includes new data relating to testicular cancer which reinforce the understanding that tumorigenic cells shape their microenvironment through cytokine actions. Clinical implications in pathologies relating to local inflammation and to immunotherapies are discussed.

## Introduction

### The Contributions of Cytokines to Testicular Functions and Health

Male fertility requires the production of functional sperm in sufficient numbers to achieve fertilization. Although the means by which this is accomplished varies between species, the processes essential for emergence of a haploid gamete with the capacity to fertilize an oocyte and contribute to healthy offspring are highly conserved. Germline development *in vivo* is dependent on the environment formed by somatic cells and the differentiation cues they provide. Knowledge of how somatic and germline cells interact is central to achieving biomedical goals relating to restoring, preserving, or restricting fertility in humans. Technical challenges related to understanding the dynamic and complex signals restrict progress toward these outcomes and have also hampered efforts to establish *in vitro* gametogenesis.

This review highlights the importance of cytokines in testis development and function that relate generally to fertility and pathology. The definition of cytokines as short-acting, short-lived signaling molecules that regulate cell functions is used here, including those that signal through JAKs and are regulated by SOCS and those utilizing other pathways, such as the MAP kinases (MAPKs). Particular areas of current research interest are highlighted relating to the likely roles of immune cells in testis development and disease. This includes new data relating to testicular cancer which reinforce the understanding that tumorigenic cells shape their microenvironment through cytokine actions.

### Cellular Architecture of the Testis

**Figure 1:**
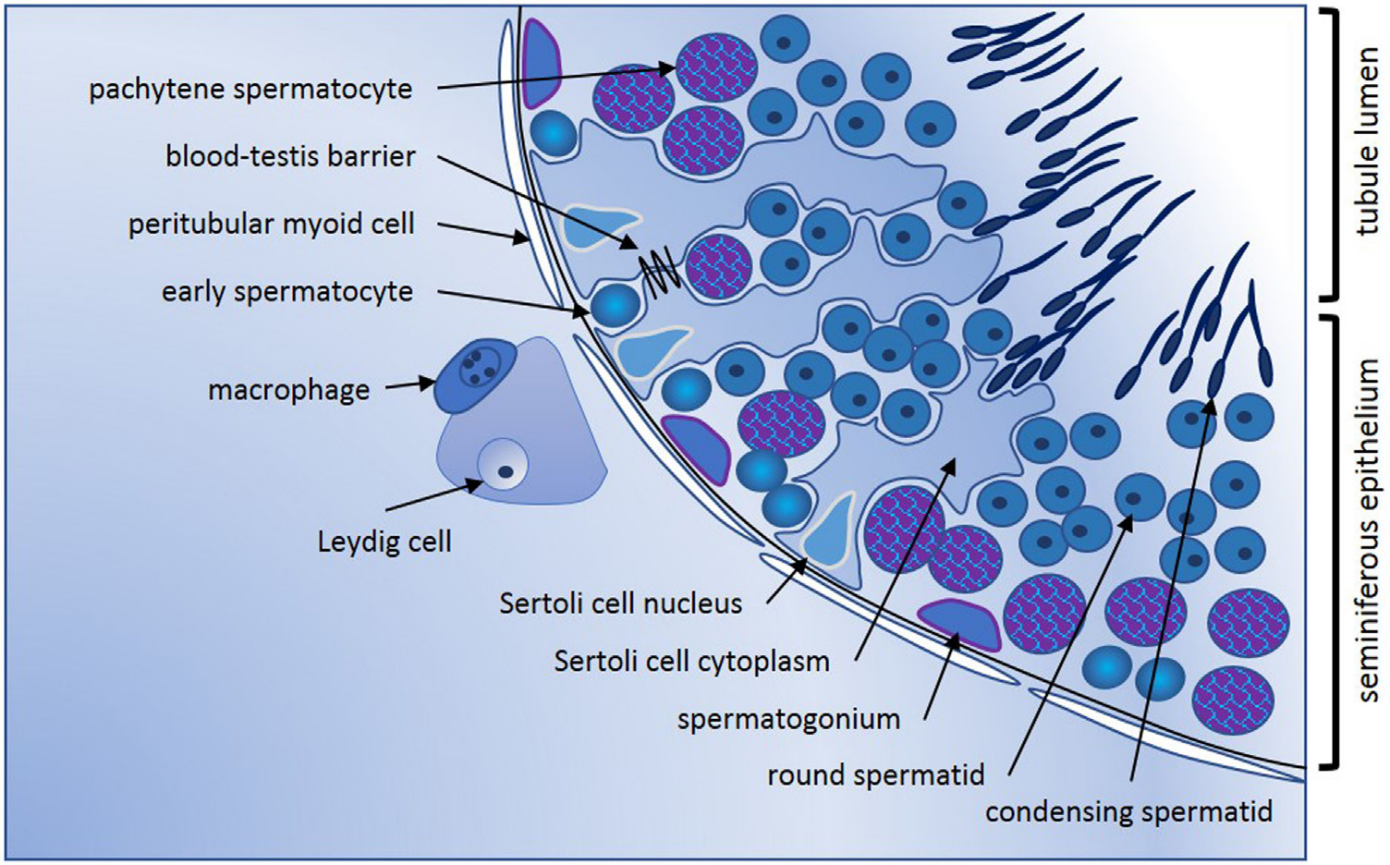
Seminiferous epithelium illustrating spermatogenic progression and indicating the key cell types. Formation of spermatozoa occurs within the seminiferous epithelium of the adult testis, which is formed by columnar Sertoli cells. Every stage of spermatogenic cell, from the least mature spermatogonia at the base, to the haploid elongating spermatid, is closely connected to or embedded within the Sertoli cells that create the epithelial architecture and provide nourishment and maturation cues essential for tight regulation of male germline maturation. Tight junctions between adjacent Sertoli cells first form the blood-testis barrier at puberty with the onset of meiosis and are vital for maintenance of spermatogenesis. Sertoli cells form the lumen of the seminiferous tubules *via* apical secretion, creating a passageway for release and transport of spermatozoa into the epididymis for additional maturation that is essential to fertility. Completely surrounding the tubules are one or more continuous layers of peritubular myoid cells. These have contractile properties that facilitate spermatozoal movement to excurrent ducts, they collaborate with Sertoli cells to generate the basement membrane which spermatogonia contact, and they synthesize other factors that influence germline development and testis function. Progressively maturing spermatogenic cells move from the tubule base toward the lumen; cells entering meiosis (early spermatocytes) lose contact with the basement membrane and pass through the Sertoli cell tight junctions into the apical compartment of the epithelium. Late stage spermatocytes complete the two meiotic divisions to become the haploid spermatids that transform from round to elongated spermatids that develop the microtubule-based tail and a head containing the highly compacted chromatin and anterior acrosomal vesicle present in spermatozoa. Leydig cells are the most abundant cell type in the interstitium; they produce testosterone that is vital for spermatogenesis and for male secondary characteristics. Blood and lymphatic vessels serve as conduits for hormonal signals that regulate testicular functions, while macrophages and mast cells are found both in close proximity to Leydig cells between the tubules, and closely opposed to peritubular cells.

Conventionally, the adult mammalian testis is considered to produce two key products, sperm, and testosterone. These are synthesized in structurally distinct compartments, the seminiferous tubules and the interstitial space [Figure 1; for comprehensive review, see Ref. (1)]. Sertoli cells form the structural platform of the seminiferous tubules within which all stages of spermatogenesis occur. The tubules are completely surrounded by peritubular myoid cells, which together with Sertoli cells synthesize a basement membrane upon which sperm precursor cells, the mitotic spermatogonia, reside. Immune cells, especially a subset of macrophages and, in human testes, a few scattered mast cells, are also found in close apposition to the tubule perimeter. Testosterone is produced by the Leydig cells, which reside in the interstitium, in close apposition to immune cells, including macrophages, fibroblasts, and both lymphatic and blood vessels. In adult animals, the mitotically dividing and progressively maturing germline precursor cells, spermatogonia, transition through meiosis as spermatocytes and develop into haploid spermatozoa, continuously embedded within the seminiferous epithelium formed by post-mitotic, columnar Sertoli cells. The least mature, mitotic spermatogonial stem cells (SSC) and their differentiated progeny are located at the base of the seminiferous tubule in post-pubertal animals, with progressively more mature germ cell types found moving toward the tubule lumen (Figure 1). Tight junctions between adjacent Sertoli cells form first at the onset of puberty, marking the end of the rapid increase in somatic cell populations. These junctions separate post-meiotic germ cells (spermatids) from the immune cells present in peri- and inter-tubular (interstitial) spaces, preventing immune cell recognition of these “developmentally late” reproductive cells as foreign.

### The Complexity of Cytokine Signaling Pathways in the Testis: Many Advances and Many Knowledge Gaps

Much of our knowledge of cytokine structure, signaling pathways, and function relates to their involvement in immune cell activity. While many factors, such as interleukins and tumor necrosis factor (TNF), control immune cell function within the testis, they also are produced by “non-immune cells” to stimulate and maintain spermatogenesis. The initial interest of testicular biologists in the roles of cytokines arose with the discovery that rat testicular extracts contained interleukin-1 (IL1), which was then shown to be produced by Sertoli cells rather than by intra-testicular leukocytes (2, 3). This discovery provided the first indication that an inflammatory cytokine, traditionally associated with immune reactions, was produced by a somatic cell of the male reproductive system when there was no evidence of immune stimulation. It subsequently became apparent that many so-called pro-inflammatory and immunoregulatory cytokines were being produced by testicular somatic and spermatogenic cells, both under normal conditions and in response to inflammatory stimuli (4).

Several interleukin family cytokines, such as interleukin-6 (IL6) and IL10, signal through cell surface receptors that feature intracellular binding sites for JAK family kinases. Upon ligand binding, the phosphorylation of STAT proteins mediates signal transduction to the nucleus to effect changes in gene activity. STAT recruitment and activation are controlled by cytoplasmic SOCS proteins. Other cytokines share the capacity for short-acting, short-lived signaling and are essential for male fertility but do not signal through the JAK/STAT pathway. These include IL1, which signals *via* the adapter protein MyD88 to activate the inflammatory transcription factor, NFκB, and members of the transforming growth factor-beta (TGFβ) superfamily, such as TGFβs and activins, which signal through serine/threonine kinase receptor subunits to activate SMAD transcription factors. Several of these cytokines were first isolated and characterized as reproductive hormones and their roles in immune signaling were discovered later. Chemokines involved in core developmental events, including CXCL12, are also essential for specific stages of germline maturation, as described below. Signaling pathway cross-talk is increasingly recognized to influence signaling outcomes [e.g., Ref. (5, 6) and reviewed in Ref. (7)], and although their specific relevance to testicular physiology is poorly understood, this is an area of intense current interest. For many cytokines present within the testis, fundamental knowledge regarding their sites of synthesis, target cells, and what regulates their production and activity remains to be revealed.

## Cytokines in the Normal Testis

### Diverse Cytokines and Functions Are Essential to Support Germ Cells: Which Ones Matter?

Important aspects of normal testis development and function are modulated or driven by cytokine activities. There is accumulating knowledge of immune cell-associated cytokines essential for fertility that maintain testicular homeostasis within the complex and dynamic environment of the seminiferous epithelium. Because cytokines are key mediators of immune cell function, and because the testis is a tissue in which cytokine functions are tightly regulated to protect and allow sperm production, there is a strong motivation to identify those present in the testis and to determine how they work. It is also important to recognize that cytokine production from non-immune cells is central to normal adult testis functions.

For example, both Sertoli and Leydig cells can be stimulated to produce large amounts of the immunoregulatory cytokine, IL6, driven at least in part by endogenous IL1. Both IL1 and IL6 are able to regulate Sertoli cell and spermatogenic cell development (4). Spermatogenic cells produce TNF. It has dual actions as a signaling molecule, to regulate Sertoli cell function and cell death, in response to toxic insults, and this activity is largely determined by the receptor with which it interacts (8).

Several members of the TGFβ superfamily provide developmentally regulated and cell-specific signals essential for normal testis development and fertility [reviewed in Ref. (9, 10)]. The activin A homodimer was identified on the basis of its formation from the βA subunits of the gonadal hormone inhibin. It was shown to be a testicular cytokine around the same time as IL1, but it took many years for this product of Sertoli and other somatic cells to be recognized as also serving immune modulatory functions. The canonical members of the superfamily, TGFβ1–3, are involved in many aspects of inflammation and immunoregulation (11), and like activin are produced by several testicular somatic cell types to regulate germline and somatic cell growth and activity (9, 12–15).

Colony-stimulating factor-1 (CSF1) and macrophage migration inhibitory factor (MIF) are developmental cytokines with central roles in macrophage recruitment and functional maturation. They are most closely associated with regulating macrophage and Leydig cell development within the testis; CSF1-deficient mice, although fertile, have severe hypospermatogenesis and are androgen deficient due to poor development of the testicular macrophage and Leydig cell populations (16, 17). The role of CSF1 in spermatogonial maintenance has been demonstrated in the adult mouse testis (18).

Interferons (IFN; α, β, and γ) are produced by many testicular cells, particularly somatic cells, and especially during viral infections. While these are implicated in protecting the testis from virus infection, there is evidence that interferons regulate Sertoli cell and Leydig cell function as well (19, 20). The first chemokine identified in the testis was MCP-1/CCL2, which is implicated in regulation of the large testicular macrophage population (21, 22).

With the diversity of signaling molecules simultaneously present within the seminiferous epithelium, what are the mechanisms by which both maintenance and continuous maturation of stem cells into spermatozoa are supported? The cycle of the seminiferous epithelium is the basis for ongoing sperm production, in which spermatogenic development is linked vertically within a Sertoli cell and conserved subsets of differentiating germ cells are observed in each cross section of a seminiferous tubule. Several cytokines, including IL1, IL6, TNF, and activin A, are produced by the Sertoli or spermatogenic cells in a cyclical manner during the maturation cycles of the seminiferous epithelium, suggesting that their actions are central to regulating this fundamental feature of testicular functions (23). Their detection in distinct seminiferous tubule cross sections of the adult testis reflects their temporally regulated synthesis and function. Other data also indicate that they serve key roles in inter-compartmental intercellular communication, regulation of steroidogenesis, and immunoregulation, including immune privilege, while also contributing to pathophysiology and the detrimental effects of inflammatory responses on testis function. Many of these aspects have been previously reviewed (23–25). In the following sections, some key examples of developmental processes and pathologies of the testis that are influenced by specific cytokines, but which have received less attention to date, are provided.

### Cytokine Signaling in Germline Cells

#### JAK/STAT Signals in Testicular Stem Cells

The vital contribution of JAK-STAT signaling to *Drosophila* germline stem cell maintenance occurs within the somatic stem cells that form the germline niche. Activation of this pathway promotes endogenous production of its inhibitor, SOCS36E, restricting growth of the niche cells (termed “the hub”) and thereby enabling spermatogonia stem cells to survive (26). The level of this signaling provides the numerical balance between somatic and germ cells, because both require access to integrins in the basement membrane for survival. Thus, appropriate pathway activation is required to create the germline:niche cell ratio required to support spermatogenesis and normal fertility. Central to this process is regulation of SOCS36E in the niche cells, which is involved in controlling downstream MAPK activity (27). Additional mechanisms implicated in control of JAK–STAT signaling within germline cells involve Wnt-2 and a H3K4me3 histone demethylase, encoded by *lid* (28–30). An important outcome of pathway activation in the germline is enhanced male stem cell survival. Mediated by the downstream target, DIAP1 (31), this includes sex-specific activation of a male gene, *Phf7*. Intriguingly, inappropriate *Phf7* expression in females is associated with the formation of germline tumors (32). These and other studies from the Matunis laboratory (33, 34) demonstrate that the impact of this pathway, which is central to fertility, is manifest in multiple cell types and in different stages of testicular development in the fly.

Less information specific to Jak/Stat signaling is available from mammalian studies. Experiments using rodent primary testicular cells indicated JAK–STAT recruitment occurs in gonocytes of the newborn testis. Pathway activation *via* a cadherin isotype was implicated in the earliest stages of spermatogenesis, when male germ cells first enter the SSC and/or differentiation pathway (35), but details are limited. In Leydig cells, JAK–STAT recruitment was documented following erythropoietin activation (36). The crucial role of these signals in the fly testis provide a rationale for future investigations assessing conserved functions for JAK–STAT signaling in both germline and somatic cells of the mammalian testis. These early observations have paved the way for explorations involving data integration from transcriptional and proteomic profiling of testicular cells to determine the expression profiles of particular cytokines and, therefore, their likely sites and timing of action.

#### CXCL12 Control of the Male Germline

##### CXCL12 Acts on Primordial Germ Cells (PGCs)

CXCL12 is a chemokine produced by somatic cells that influences early mammalian germline cells, including at stages considered of relevance for the initiation of testicular germ cell tumors (TGCTs). CXCL12 is produced as six different isoforms in humans, and different functions have been ascribed to these [reviewed in Ref. (37)].

Primordial germ cells and gonocytes both express the CXCL12 receptor, CXCR4, a seven-transmembrane protein which signals via G-proteins, leading to MAPK activation and to engagement of the phosphoinositol-3-kinase pathway. Somatic cell-production of CXCL12 facilitates the later stages of PGC migration into the genital ridge immediately prior to their sexual differentiation, and upon reaching the nascent gonad, CXCL12 supports gonocyte survival (38, 39). Mice lacking the CXCR4 receptor have fewer gonocytes, and the difference between wild type and mutant animals increases with developmental age, indicating that CXCL12 regulates the germline cell cycle in the fetal testis (38, 39). Studies of chick and zebrafish demonstrate this crucial function of supporting the earliest germ cell subtypes is evolutionarily conserved (40).

##### CXCL12 Influences SSC Fate

CXCL12–CXCR4 signaling is also central to mouse SSC fate (41, 42). The receptor is present *in situ* in the subpopulation of spermatogonia expressing markers of undifferentiated spermatogonia, and CXCR4 function is required for maximal colonization by spermatogonia transplanted into recipient gonads to assess their stem cell activity. In addition, *CXCR4* transcript levels correlate directly with stimulation by glial cell line-derived growth factor (GDNF) and fibroblast growth factor 2 (FGF2), factors that sustain SSC functionality. Evidence that this signaling pathway in SSCs affects cell adhesion to the basement membrane was provided from a range of elegant transcriptome profiling, cell culture, and cell adhesion assays (41, 42), analogous to its functions in other stem cell systems.

As described above, PGCs and gonocytes express the CXCR4 receptor. In addition, the CXCR7 receptor protein that also binds CXCL12 is present on undifferentiated spermatogonia (43). Its specific role in germ cells is unclear, as CXCR7 serves variably as a decoy receptor and as a signaling receptor, to enhance and restrict cell responses to CXCL12 in different contexts [reviewed in Ref. (44)]. Evidence that all of these components perform conserved functions in the testis is provided from their expression profiles in the marmoset and human, which mirror those in the mouse (45).

An additional cytokine that can signal through CXCR4 is MIF (37, 46). This widely expressed chemokine is often linked with pro-inflammatory events because of its role in the innate immune system, but it has also been linked with regulation of B lymphocyte migration (46). Early investigations of MIF in the rodent testis identified it as a Leydig cell product that could act on both Sertoli and peritubular cells to regulate their signaling (47, 48). Intriguingly, its production appeared to switch on in adult rat Sertoli cells *in vivo* when a toxicant was administered to achieve acute depletion of Leydig cells (47). A recent investigation of MIF function suggests that it may contribute to regulation of spermatogonial migration (49), which would be in accord with results of studies demonstrating this function for CXCR4 signaling (41, 42).

### Cytokines Function in Immune Regulation to Protect Germ Cells

#### Immune Privilege in the Testis

**Figure 2:**
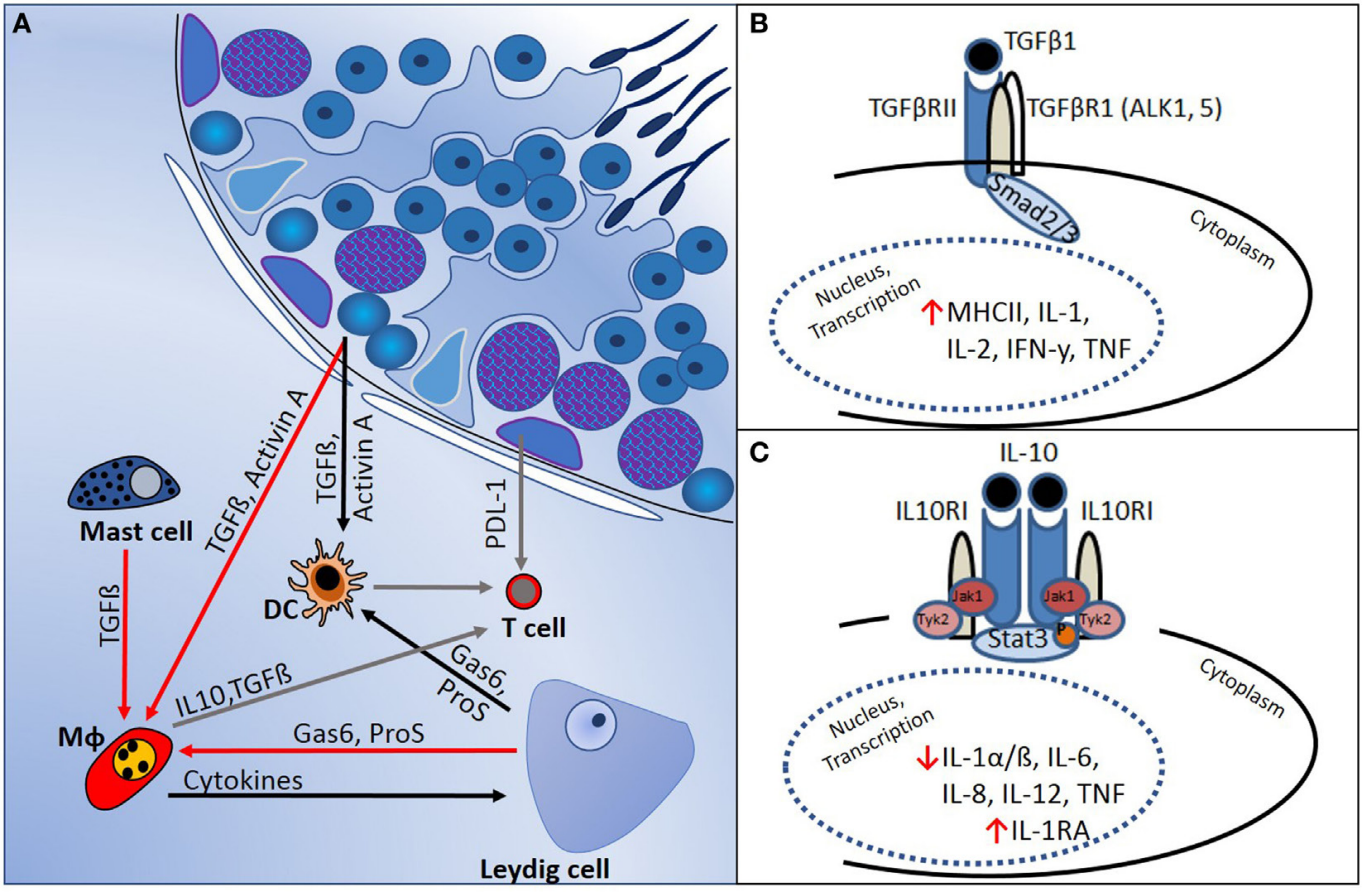
Regulation of immune responses in the testis is mediated by a combination of structural and cellular-derived factors. **(A)** Production of cytokines and other immunoregulatory molecules by interacting cell types [mast cells, dendritic cells (DC), T cells, macrophages (Mφ), and Leydig cells] in the testicular interstitial space creates an immunoregulatory environment, as outlined in the text. **(B)** TGFβ-signaling *via* Smads 2 and 3 suppresses pro-inflammatory cytokine production and reduces cell-mediated immunity. **(C)** IL10 actions, mediated *via* JAK–STAT signaling, inhibit pro-inflammatory cytokines and induce anti-inflammatory responses (e.g., IL1RA production).

In several mammalian tissues, such as the brain, eye, pregnant uterus, and testis, it is necessary to prevent immune responses against proteins that are normally hidden or are expressed late during development and which might become immunogenic autoantigens. The concept of “immune privilege” in these tissues relates to the mechanisms that underpin regulation of local cell function to prevent pathogenic autoimmunity (50). Haploid germ cells in the testis, in particular, express proteins that not subjected to the normal mechanisms of central tolerance, and strong adaptive immune reactions against these antigens can develop. This immune regulation is supported by the physical segregation of haploid germ cells, which develop only after puberty, away from direct contact with immune cells, as they become progressively more apically positioned within the seminiferous tubule epithelium (Figure 1). Sertoli cells actively phagocytose the residual haploid germ cell cytoplasm upon release of spermatozoa into the tubule lumen, and they have an important immunoregulatory function (51). Leydig and Sertoli cells support the immune privileged state by production of immunosuppressive molecules that include activin A, testosterone, PDL-1, Gas6, ProS, and TGFβ (Figure 2) (11, 50–52). In addition, certain germ cell types express FasL, which can bind to the Fas receptor, expressed by T-lymphocytes. This interaction may induce lymphocyte apoptosis to avoid cell activation (53). Immune cells within the healthy testis, most likely macrophages, are key contributors to the immunosuppressive milieu through their production of anti-inflammatory cytokines, such as IL10 and TGFβ (54, 55), as discussed below. Without this control, presentation of testis-specific antigens on the surface of testicular macrophages and dendritic cells (DC) can lead to activation of T-lymphocyte responses and cellular or humoral immune reactions.

#### Testicular Immune Cells under Physiological Conditions

The normal mammalian testis contains diverse immune cells within the interstitial compartment, comprising resident macrophages, T-lymphocytes, DC, and mast cells (56–61). The numbers and distribution of immune cells differs between mammalian species, potentially reflecting differences in lifespan, functional activity, and exposure to pathogens.

The most abundant immune cell subtype in the testis is interstitial resident macrophages (62), most of which are physically closest to the interstitial Leydig cells (63, 64). Testicular macrophages arise from yolk-sac hemopoietic progenitors and are localized to the mesonephric region near the gonad border during early gonad sex differentiation (E10.5–E12.5) (65). They are essential for normal vascularization and testis cord formation in the fetal testis as well as Leydig cell development in the neonatal testis (17). In adult testes, transient depletion of testicular macrophages in a mouse model led to 75% reduction in total spermatogonial precursor number, revealing their vital importance in the regulation of the SSC niche (66).

Testicular macrophages comprise a heterogeneous population in the adult, based on cell-specific markers. Distinct interstitial macrophage subsets have been identified in the human and rat, which differentially express CD68 (a lysosomal glycoprotein) and CD163 (a cell surface glycoprotein member of the scavenger receptor cysteine-rich superfamiliy) (56, 58, 61, 67, 68). In general, macrophages have highly variable properties and can either develop into M1 macrophages, which are classically activated and pro-inflammatory, or into M2 macrophages, which are alternatively activated and anti-inflammatory (69, 70). In mice, pro-inflammatory stimuli, such as bacterial lipopolysaccharides and IFNγ, lead to macrophage polarization toward M1 (70–72), whereas anti-inflammatory/immunoregulatory stimuli, such as IL4, IL10, IL13, TGFβ, and glucocorticoids, polarize toward an M2 subpopulation [(73–76); reviewed in Ref. (77)]. The majority of testicular macrophages detected in rodents have an M2 phenotype (54, 68) and play important roles in maintenance of normal tissue homeostasis, fetal development, and immune response regulation. However, inflammation initiated by microbial pathogens or other immune events can also stimulate them to release pro-inflammatory cytokines, such as IL1, IL6, TNF and CCL2 (22, 70, 71, 78).

In healthy testis tissue, normal germ cell development is not impaired by the presence of the immune cells and their cytokines. Conversely, a change of immune homeostasis, associated with infection and inflammation, can lead to male infertility. In testicular germ cell neoplasia, there is strong evidence that the immune cell environment is severely disrupted. Respective pathological conditions are discussed in Sections “Cytokines in Testicular Pathologies” and “Knowledge of Testicular Cytokines Used to Address Key Clinical Issues.”

## Cytokines in Testicular Pathologies

### Testicular Cancer

#### The Origin of TGCTs and Research Challenges

Germ cell neoplasia *in situ* (GCNIS) cells are the pre-malignant precursor cells of TGCTs, the most common testis tumor type. They develop within the testicular niche from PGCs or their immediate male progeny, gonocytes, which fail to mature into spermatogonia [reviewed in Ref. (79)]. This outcome is part of the spectrum of disorders associated with an elevated risk of infertility, termed the testicular dysgenesis syndrome, or TDS (80). GCNIS cells are large and round, with a sparse cytoplasm; they are morphologically similar to gonocytes and express markers of primitive germline cells, including OCT4, NANOG, GDF3, KIT, and AP-2γ. Their transformation into neoplastic cells appears linked to the hormonal changes of puberty, with the non-seminoma subtype typically detected in men between 17 and 30 years of age and seminomas more commonly identified in 25- to 40-year-olds. Seminomas are morphologically similar to GCNIS cells and express the same markers. A striking feature of these tumors in many patients is the presence of immune cell infiltrates, discussed below. Non-seminomas are heterogeneous, containing various differentiated tissue and cell types, undifferentiated embryonal carcinoma cells and cells resembling extra-embryonic tissues; this subtype is more aggressive and clinically challenging.

The failure of early germ cells to differentiate, which is considered to underpin GCNIS formation, is thought to relate to a somatic environment deficiency potentially arising from genetic and/or environmental factors (79, 80). The subsequent development of GCNIS cells into either seminoma or non-seminoma TGCTs reveals their cellular plasticity. However, the mechanisms underlying GCNIS establishment and progression into either subtype are largely obscure, particularly because this process may take years to decades. Genome-wide association studies (GWAS) have most frequently identified *KITL* and *KRAS* alleles as the highest genetic risk factor (81), providing clues to processes or events that may enable inappropriate survival of germ cells that do not differentiate. Mice and other common laboratory animals do not develop TGCTs similar to those found in humans; the timespan from PGC formation to onset of spermatogenesis in a mouse is less than one month, while in humans this transformation typically lasts several years. As a consequence, studies addressing human GCNIS etiology, transformation into frank TGCTs, and mechanisms of metastasis are ethically and practically limited by the paucity of research materials. Although animal models inform us about fundamental processes of germline differentiation, important progress toward understanding testicular neoplasia requires access to patient samples, including archival and fresh specimens and derived cell lines.

#### What Cytokines Contribute to the Emergence and Differentiation of TGCTs?

Knowledge of rodent spermatogenesis (summarized in Section “CXCL12 Influences SSC Fate”) led to the hypothesis that aberrant CXCL12 signaling may contribute to the “dedifferentiation” of PGCs into GCNIS cells. CXCL12 is known to mediate normal cell migration and is implicated in many neoplasias, including the overexpression of its canonical receptor, CXCR4, in various cancers (5, 6). The proposed involvement of CXCL12 in GCNIS to seminoma progression was based on the observation that the *CXCR4* transcript level was generally higher in human seminoma samples than in those from healthy controls, whereas *CXCL12* did not differ (82). Variable *CXCR4* levels between testis tumor samples suggested responsiveness to CXCL12 may differ between individuals. However, no substantial differences in CXCL12 and CXCR4 protein staining was detected by immunohistochemical analyses of normal and pathological testes, and no apparent phosphorylation differences could be identified to implicate MAPK (MEK1/2, ERK1/2), STAT3, AKT, or ERK in pathway signaling. The human seminoma-derived cell line, TCam-2 (83) was also used by McIver to investigate CXCL12 signaling mechanisms and outcomes relevant to TGCTs. Although no significant differences in signaling molecule phosphorylation status, proliferation, or migration were measured, CXCL12 exposure increased TCam-2 cell invasion in a chamber assay (82). While the role and impact of CXCL12 signaling for TGCTs *in vivo* is unresolved, it remains a key candidate for further investigation. Studies examining the atypical receptor, CXCR7, and alternative CXCR4 ligands such as MIF may be required to ascertain the contributions of this pathway to testicular neoplasia.

Several highly significant findings implicate abnormal TGFβ superfamily signaling in TGCT biology. A zebrafish model arising from genetic disruption of BMP signaling (inactivating mutation in the type IB BMP receptor, encoded by *alk6b*) exhibited impaired spermatogonial differentiation and overgrowth of undifferentiated germ cells, analogous to events considered to underpin human TGCT initiation (84). Examination of Smad1/5/8 in human germ cell neoplasias showed that the less differentiated subtypes, including seminomas, lacked nuclear SMAD 1/5/8 which indicates the absence of canonical BMP signaling; by contrast, more differentiated tumor types display this signaling (84, 85). The potential involvement of activin A signaling in TGCTs was demonstrated through immunohistochemical localization of activin type I (ALK2 and ALK4) and type II receptors (ActRIIB) in GCNIS, seminoma and non-seminoma cells (86). Nuclear phospho-SMAD2/3 signal was present in all seminoma samples, indicating active activin/TGFβ signaling in these tumors, however, a subset of seminoma specimens exhibited expression of betaglycan or the inhibin alpha subunit, both of which block activin signaling and are normally only produced in somatic cells (87). These observations highlight the likelihood that individual tumors have different TGFβ signaling activities. Furthermore, elevated TGCT risk was associated with a SNP in the *INHA* gene, which encodes the inhibin alpha subunit (88), though its relationship to altering activin bioactivity remains to be elucidated. This is an interesting avenue to address, because the bioactivity levels of activin in the developing mouse testis have a great impact on germ and somatic cell function [reviewed in Ref. (9, 10)].

*In vitro* studies addressing the impact of TGFβ superfamily signaling on testicular tumors have explored its potential as a target to restrict testicular tumor growth. An organ culture strategy was applied using small fragments (1 mm^3^) of primary tumor materials in 30 µl hanging drop cultures (89), for parallel analyses of molecular and histological outcomes, with maintenance of cell viability and proliferation for at least 7 days in normal and neoplastic human testis samples (90). GWAS identified that *KITL* polymorphisms represent one of the highest risk factors for testicular neoplasia (91, 92), thus regulation of this signaling pathway may be a logical target for therapeutic intervention. The KIT Ligand (encoded by *KITL*, also known as Stem Cell Factor or SCF) is produced by Sertoli cells in the testis, and it signals *via* the KIT receptor tyrosine kinase that is found on germ cells at specific developmental stages (PGCs, gonocytes, differentiated spematogonia) and Leydig cells (all ages) to regulate their functions. Activin A bioactivity regulates synthesis of KIT and/or KITL, including in the testis (93–96), and hanging drop culture of testicular fragments in Activin A resulted in downregulation of both KIT mRNA and protein in a seminoma sample (90). The specific impact on KIT signaling in seminoma cells has yet to be addressed. This culture approach could be used for patient-specific testing of treatments and offers a powerful investigative tool for studying cytokine impact in the intact cellular milieu of TGCTs.

The TCam-2 seminoma cell line has enabled important mechanistic investigations of TGFβ superfamily member functions. Exposure to BMP4 and retinoic acid increased activin receptor transcript levels (*ACTRIA, ACTRIIA*, and *ACTRIIB*) and enhanced TCam-2 cell survival and/or proliferation, whereas activin A reduced *ACTRIB* and did not affect overall survival or proliferation (97). Factors that support human embryonic stem cell differentiation into the endodermal lineage, namely FGF4 and its co-factor heparin, induced the expression of mixed non-seminoma tumor cell markers in TCam-2 cells (98). A combination of TGFβ1, FGF4, and EGF could mediate this outcome, and intriguingly, a BMP signaling blockade was identified as a potential initiating event, based on SMAD phosphorylation patterns. These findings from independent laboratories implicate TGFβ superfamily signaling as central to controlling TGCT cell behavior, supporting the concept that dysregulated TGFβ superfamily signaling may elevate the risk of TGCT emergence.

These outcomes relate to the impact of changing cytokine levels and functionality within the tumor microenvironment on tumor cell fate and differentiation. In this regard, the commonly observable dichotomy of cytokine actions may be of particular relevance. TGFβ1 is one example of an ambivalent cytokine. It is classically regarded as anti-inflammatory, anti-tumorigenic and anti-proliferative, but may under certain conditions exhibit contradictory effects, both oncogenic and tumor-suppressing, depending on the cellular context (99) As these culture studies indicated, activin A or TGFβ1 signaling in testis cancer may restrict seminoma growth by promoting cellular differentiation, thus further efforts to elucidate the functional interplay of these ligands with other cytokines in the tumor microenvironment will be informative.

#### Cytokines As Mediators of Crosstalk between Immune Cells and TGCTs

Infiltrating immune cells are common in various tumors, including TGCTs, contributing to the milieu of cytokines and chemokines that comprise the tumor microenvironment (TME). Important crosstalk between tumor-infiltrating immune cells and cancer cells may reinforce the development of a TME which is optimal for tumor survival and expansion. As described in Section “Cytokine Signaling in Germline Cells,” maintenance of the testicular environment for production of sperm is dependent on cytokines secreted by both testicular somatic cells (Sertoli, Leydig, peritubular) and resident immune cells. Additionally, some typically “protective” cytokines may, under pathological conditions, negatively impact on testis function and physiology. Whereas many studies have classified the immune cell types that are abundant in testicular tumors, few have assessed the composition of the accompanying cytokine milieu, and hence this important characteristic of the testis cancer microenvironment is poorly understood.

T cells and macrophages are typical cellular components of testicular tumors (60); by contrast, the presence and roles of DC, as important antigen-presenting cells (APC), remains largely unknown. Under certain conditions, nascent tumor cells escape regulation by the host immune system by inducing APC malfunction, thus hijacking immune surveillance, and in turn initiating immunosuppressive cell recruitment to create a tumor-tolerant microenvironment. Zheng et al. (67) assessed the presence of DC subsets in human seminomas. Significantly higher numbers of CD11c+ myeloid DC (mDC) were found in tumor tissues compared to healthy controls, with a proportion of mDC presenting an immature phenotype, potentially associated with cancer progression. The CD11c+ mDC expressed indoleamine-2,3-dioxygenase (IDO, a regulator of T cell development), VEGF and IL4 (immune tolerance cytokines), as well as IL6 and IL23a (pro-inflammatory cytokines that can be involved in Th17 phenotype skewing) but not TGFβ1 or IL10 (frequently involved in anti-tumor immune responses). In contrast to other studies, significantly higher numbers of IL17+ (potentially Th17 cells) and FoxP3+ (regulatory T cells) cells were detected in seminomas compared to controls. This result led the authors to propose that a shift in the testicular TME toward a Th2-driven immune response, induced by IL4+ CD11c+ mDC, enables TGCT immune escape *via* immune suppression.

Bialas et al. (100) also examined both pro- and anti-inflammatory cytokines in seminomas. *IL6* and *TNFR2* transcripts were both significantly increased in testis cancer, whereas *IL10* and *TNFR1* levels remained largely similar to respective controls. A pro-proliferative effect of IL6 in testis cancer was suggested, contrasting the outcomes of *in vitro* treatments of seminiferous tubules in which IL6 was shown to induce normal germ cell apoptosis. By contrast, IL10 was proposed to maintain an immune-tolerant testicular environment. These conclusions align with knowledge of IL6 as a cell growth stimulating factor in other organs/diseases (101) and the known immunosuppressive properties of IL10 (102). However, no further examination of IL6 as a potential testis-tumor promoting factor or its underlying signaling pathways has been conducted.

Our group also studied testis cancer patient samples, combining a more comprehensive analysis of immune cell subtypes in the context of measurements of cytokine transcripts in the surrounding TME (61). This allowed a more extensive examination of immune cell subtypes and functions directly associated with measurements of the specific testis cancer cytokine milieu. Significantly higher levels of transcripts encoding pro-inflammatory cytokines IL1β and TNF matched an extensive recruitment of macrophages (CD68+) and/or DCs (CD11c+) in testis cancer samples. In seminomas, significantly increased levels of *IL2* and *IFNγ* were detected, indicating the likelihood of T cell functional polarization toward a Th1-driven immune response (103). Expression of Th2-related, anti-inflammatory cytokines in testis cancer samples was either not different (IL4) or significantly decreased (IL5, IL13, IL23a) compared to that found in non-neoplastic controls, suggesting that this cytokine milieu may dominate in physiological circumstances to maintain testicular immune privilege and normal spermatogenesis. Adding to the knowledge of the TGFβ superfamily in testis cancer, TGFβ1 was also significantly higher in testis cancer samples. The approach of combining cell type enumeration with cytokine analysis (61) also provided mechanistic insights about the prominent presence of B cells selectively in seminoma samples which had earlier led to the proposal that B cells may be pivotal to the immunopathology of testicular cancer (104). Klein measured greatly elevated transcript levels of both IL6, the most important B cell activating factor (61, 105), and CXCL13, which encodes a key factor for B cell recruitment. Moreover, a similar increase in both *IL6* and *CXCL13* transcripts was documented for two samples with localized GCNIS, suggesting that IL6 may function in the early stages of neoplastic progression.

**Figure 3:**
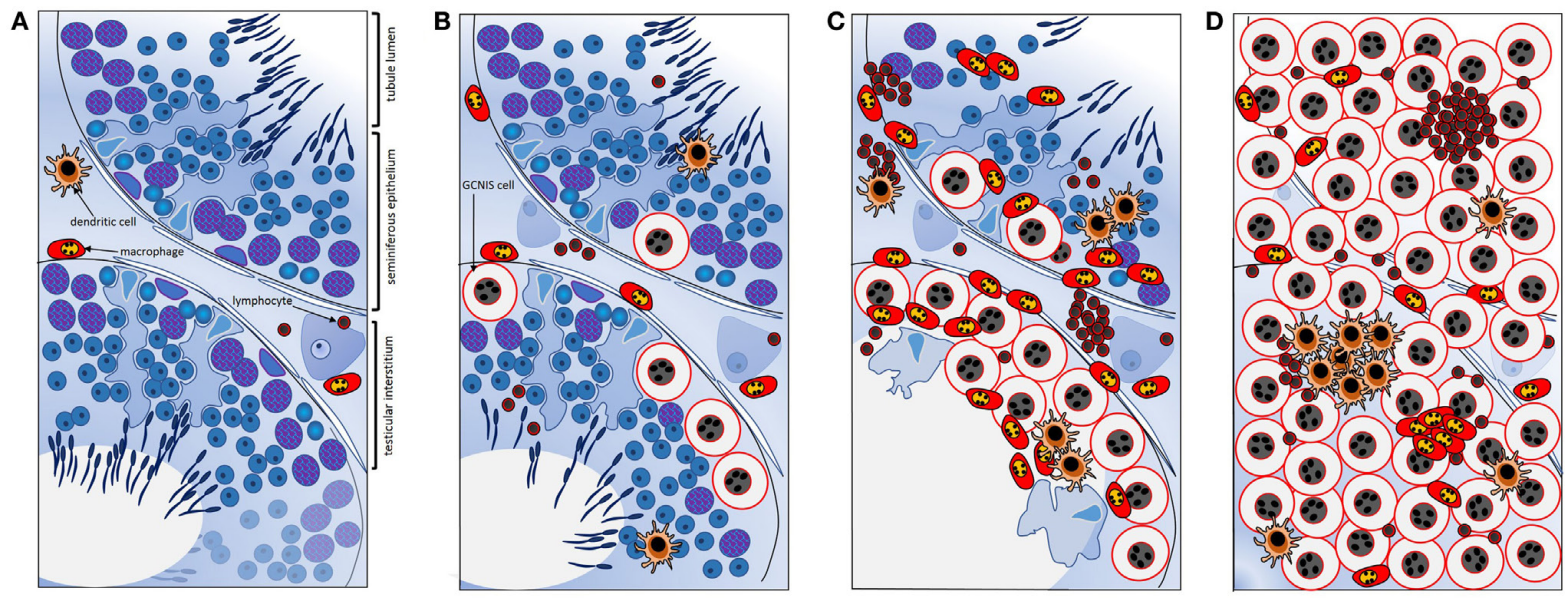
Schematic summary of key transitions documented relating to cytokines and immune cells during testicular germ cell tumor progression indicate their involvement in the progressive changes that underpin testicular neoplasia. **(A)** In normal spermatogenesis, the number of resident immune cells present in the testicular interstitium is low relative to other somatic cell types. **(B)** Tubules containing even a few GCNIS cells are associated with changes in the cytokine environment; GCNIS cells themselves may be the source of significant interleukin-6 (IL6) and other cytokines that influence immune cell behaviors. **(C)** As the number of GCNIS cells increase, different types of immune cells infiltrate the testis, and the local cytokine profile transitions to a “neoplastic pattern.” Direct contact with immune cells may enhance cytokine production by GCNIS cells, yielding an environment which facilitates blood–testis barrier disruption and tumor progression. **(D)** In seminoma, many different types of immune cells and immune cell clusters [mainly comprised of B cells and CD11c+ dendritic cells (DCs)] are present, and characteristic “neoplastic” cytokine microenvironment is detectable that likely facilitates tumor progression. IL6 and other cytokines may be crucial as maintenance and progression factors. Adapted from Ref. (107).

Further work by Klein et al. (106) created a co-culture system to examine the contributions of different cell types to the testis cancer cytokine TME, using TCam-2 cells to represent neoplastic germ cells (seminoma) and peripheral blood monocytic cells (blood-derived immune cells), as a surrogate for testis cancer-infiltrating immune cells. The cytokine pattern observed after 24 h of co-culture was similar to that documented in *ex vivo* seminoma samples (106). Intriguingly, this appeared to be initiated by the TCam-2 cells only following direct contact with immune cells, with IL6 measured at significantly higher levels in TCam-2 cells than in PBMCs recovered from the co-cultures. A model depicting the progressive changes in testicular immune cell populations during the progression from healthy, tumor-free to seminomatous germ cell neoplasia in relationship to local production of cytokines is presented in Figure 3 [adapted from Ref. (107)].

Given the cumulative data on the link between IL6 and testis cancer, an experimental immunotherapeutic approach targeting IL6 could provide knowledge of the clinical applicability of these findings. IL6 archetypically signals through the JAK/STAT signaling pathway, with STAT3 phosphorylation indicative of active signaling *via* the IL6R. Dysregulated IL6 production has been identified in several cancers, often positively correlated with tumor growth and invasive behaviors (108). The anti-IL6R antibody Tocilizumab, which blocks both membrane-bound and soluble IL6R isoforms, has undergone *in vitro* testing to evaluate its effects on cancer cell lines. Similar investigations in testis cancer primary samples or with available cell lines may yield insights regarding the potential of immune-therapeutic options for testis cancer patients.

### Inflammatory Conditions of the Testis: How Frequent and How Important Are They?

Infection and inflammation of the reproductive tract are accepted as important etiological factors of male infertility (109, 110). Testicular inflammation occurs as a complication of acute epididymitis, which affects approximately 400 per 100,000 males each year (111). In most cases, the disease is unilateral and caused by ascending infections with sexually transmitted bacteria such as *Chlamydia trachomatis* or common uropathogens such as *Escherichia coli* (112). Intriguingly, the course of epididymo-orchitis remains unpredictable despite antimicrobial therapy, with a prevalence of irreversible oligo- or azoospermia in 30 and 10% of patients, respectively (113). Orchitis as a primary *de novo* inflammatory process evolves as a complication of systemic, predominantly viral infections due to blood-borne dissemination of the pathogen [reviewed in Ref. (114)]. The classical paradigm of this type of orchitis is seen with mumps infection in post-/pubertal males and associated with the risk of testicular atrophy. Notably, a wide range of other viral infections, including Coxsackie viruses, Epstein–Barr, influenza, and human immunodeficiency viruses, can be associated with orchitis (115, 116). Recently, the sequelae of Zika virus infection, including testicular inflammation and deterioration of spermatogenesis, have been delineated in a mouse model (117); however, whether or not Zika-related, overt orchitis occurs in man remains to be elucidated. A predominantly granulomatous, chronic orchitis is known as a manifestation of tuberculosis, syphilis, lepromatous leprosy, and brucellosis (114).

In addition to infectious disease, non-infectious causes of inflammation in the testis, other than neoplasia (see section “Testicular Cancer”), must be considered. Chronic granulomatous orchitis of unknown etiology in elderly men has been classified as “non-specific” or “idiopathic” (114). Moreover, the testis can be involved in systemic autoimmune diseases such as vasculitis or lupus erythematosus (118).

Acute symptomatic orchitis is considered to be rare from the clinical perspective; however, incidents of subacute or chronic, asymptomatic testicular inflammation of infectious, post-infectious, or primarily non-infectious origin may remain largely obscure, despite important functional consequences for male fertility. For example, systematic re-examination of testicular biopsies from infertile patients revealed asymptomatic focal inflammatory lesions in approximately 30% of cases (114). In adult men who underwent orchidectomy because of cryptorchidism, focal lymphocytic infiltrates were found in over 40% of the specimens (119). These findings highlight the likelihood that pre-existent testicular disorders may be accompanied by inflammation. However, autoimmune orchitis, which can be induced in experimental animals as a vigorous organ-specific immune response by active immunization with homologous testis homogenates, has not yet been established as a clinical entity [reviewed in Ref. (24, 120, 121)].

Upon assessment of these clinical observations, it is apparent that testicular immune privilege does not preclude effective innate and adaptive immune responses to infectious pathogens or other noxae. As a hallmark of inflammatory disease of the human testis, recruitment of non-resident immune cells can be observed. Infiltrating inflammatory cells obviously overcome the immunosuppressive milieu and indicate a profound disturbance of the delicate local immune regulation (122). Apart from edema, histopathology of both acute bacterial epididymo-orchitis and viral orchitis reveals massive infiltration of the interstitial compartment and seminiferous tubules with immune cells, including neutrophils (115, 123). Perpetuated chronic (sterile) inflammation is characterized by multifocal peritubular lymphocytic infiltrates which are also encountered in testicular biopsies of infertile men. Increased numbers of immune cells (mast cells and macrophages) accumulate specifically in the seminiferous tubule walls, which frequently appear fibrotic. Affected tubules show degeneration of the germinal epithelium sparing few spermatogonia and Sertoli cells; concomitant thickening of the lamina propria may result in complete fibrosis of the tubules accompanied by increased collagen production (“tubular shadows”) (123). Notably, these are typical morphological features of experimental autoimmune orchitis (EAO) (124–127). Consistent with relevant animal models, infiltrating lymphocytes are primarily effector memory T cells (CD45R0+; CD4+>CD8+), which are accompanied by increased numbers of non-resident CD68+ macrophages and mast cells (58, 123, 128–132). In contrast to EAO or infectious orchitis in rodents, the role of CD11c+ cells (putative DCs) seen in low numbers in non-neoplastic human testicular inflammation is unclear (61, 126).

As outlined above, there is growing evidence from animal models that numerous autocrine and paracrine mediators play a dual role in the testis (24, 50, 133). Resident macrophages and mast cells as well as non-immune somatic cells such as Sertoli cells have been shown to produce a plethora of cytokines, including both pro- and anti-inflammatory molecules such as IL1β, IL6, TNFα, *IFNγ*, members of the TGFβ family, and IL10 (134–137). Compared to murine models, however, data illustrating the involvement of cytokines in human testis physiology and disease are scarce. There is suggestive evidence that maintenance of the testicular immune privilege and normal spermatogenesis are favored by a Th2-balanced cytokine milieu (IL4, IL5, IL13), in contrast to the Th1-driven immune environment of neoplasia (61). As reported by Bialas et al. (100), mRNA levels of anti-inflammatory IL10 remain largely unchanged in various testicular pathologies. Together with IL10, changes in TGFβ1 levels may reflect the presence or expansion of immunoregulatory T cell subtypes (e.g., CD4+CD25+Foxp3+Treg cells) (52, 138–140), which are yet to be identified and explored in the human testis. Moreover, exposure to Th2-related cytokines, IL10, and TGFβ polarizes testicular macrophages toward the tolerogenic M2 phenotype (54, 73). M2 macrophages themselves secrete high amounts of anti-inflammatory (IL10, TGFβ) and low levels of pro-inflammatory cytokines (IL12, TNF) (54, 141).

Testicular inflammation obviously occurs despite the presence of immunoregulatory cells and anti-inflammatory cytokines. In this context, IL23a-producing CD68+ and CD11c+ cells most likely orchestrate Th17 cells, detected using immunohistochemistry along with their key product IL17a in testis biopsies from infertile men with signs of inflammation (131, 142). We have observed increased levels of pro-inflammatory cytokine mRNAs, such as those encoding IL1β, TNF, and *IFNγ*, in selected specimens with disturbed spermatogenesis and inflammatory lesions using PCR (61). In contrast to neoplasia, overall quantitative analysis revealed only minor differences between biopsies with hypospermatogenesis/focal infiltrates and normal testes, and somewhat conflicting results were also obtained for *IL17a* transcript levels (61). This may reflect the inherent heterogeneity of human testis biopsy samples and the highly variable distribution of focal cell populations.

With regard to the recruitment, trafficking, and activation of leukocytes to the site of inflammation, chemokines, chemokine receptors and adhesion molecules are decisive mediators. In murine EAO, upregulation of cell adhesion molecules (CD31, CD44, CD106), in conjunction with increased levels of chemokines (MCP-1, macrophage inflammatory proteins 1α and 1β) and chemokine receptors (CCR2, CCR5), contribute to the formation of a chemotactic gradient within the testis, causing characteristic peri- and intratubular leukocyte infiltration (136, 143). DCs isolated from EAO rat testes showed upregulated expression of CCR7, which is responsible for the migration of these APC to the draining lymph nodes (144). Together with IL6, MCP-1 was also described as a factor released by human testicular peritubular cells upon activation of TNF receptors (145).

In a cross-platform gene expression analysis of testicular biopsies with spermatogenic failure, Spiess et al. (146) identified an increase of transcripts encoding the high-affinity IgE receptor and the mast cell-related fractalkine receptor (CX3CR1). This observation is in line with increased numbers of mast cells consistently found in testicular biopsies from infertile men with impaired spermatogenesis (61, 129, 132). In addition to pro-inflammatory cytokines, such as TNF and IL6, mast cells produce the serine protease tryptase, which may act as a potent mitogen for fibroblasts and peritubular cells resulting in enhanced synthesis of collagen and subsequent tubular fibrosis (59, 147, 148). Activation of the tryptase receptor proteinase-activated receptor-2 on peritubular cells *in vitro* led to expression of MCP-1, cyclooxygenase-2, and TGFβ2 (149).

Besides cytokines and chemokines, other pro-inflammatory molecules, such as high-mobility group box protein 1, are involved in the regulation of inflammatory reactions in rat and human testis and might serve as targets for therapeutic intervention (150). In addition, involvement of galectin-1, activins, and inhibin in the development of testicular immunopathology is documented (127, 151–153).

There is compelling evidence that inflammatory infiltrates are associated with disruption of the blood–testis barrier in affected seminiferous tubules (120, 133). As a key pro-inflammatory cytokine in testicular immunopathology, IL6 has been described to perturb the integrity of the blood-testis barrier in rats (154). Recent results indicate that IL6 acts through inhibiting protein degradation or activating phosphorylated ERK in Sertoli cells (155). *Via* the transcription factor Zfp637 it can directly affect spermatogonia and, thus, interferes with germ cell differentiation or degeneration. Along with IL6 and its receptor, TNF/TNFR1, Fas/FasL and Bax/Bcl-2 systems have all been shown to be involved in germ cell apoptosis in rat EAO (78, 144, 156). Among tight junction proteins in the seminiferous tubules, the highly conserved claudin-11 may represent a putative target (157).

## Knowledge of Testicular Cytokines Used to Address Key Clinical Issues

### Cytokines As Non-Invasive Diagnostic Tools

Cytokines and chemokines produced by resident immune cells and non-immune somatic cells in the testis are also found further downstream in the reproductive tract. The vast majority have been detected in human seminal plasma, and pro-inflammatory molecules such as IL6, IL8, and TNFα are routinely used as markers of male genital tract inflammation (158). Taking the composition of human semen into consideration, however, changes in seminal cytokine levels are not compartment-specific and do not necessarily reflect testicular (immuno-) pathologies (110, 159). As an example, KITL has been proposed as testis-specific seminal marker for spermatogenesis (160). Ligands in the TGFβ superfamily, inhibin B and anti-Mullerian hormone, produced by Sertoli cells are typically used to assess testicular function, as their production reflects the presence or absence of ongoing spermatogenesis (161, 162).

With regard to the high expression of IL6 associated with testicular germ cell neoplasia (i.e., seminoma), the possible diagnostic (and/or prognostic) value of corresponding levels in seminal plasma and/or peripheral blood remains to be elucidated (61, 106). Proteomics of testicular fluids characterizing different pathologies (163) may help to identify new organ-specific immunoregulatory molecules or diagnostics to indicate the level of function within the seminiferous epithelium; these may also occur in seminal plasma and/or peripheral blood. Moreover, testis-specific molecular signatures could be developed by combining different cytokines and related molecules in a multiplex assay approach.

### Cytokines As Therapeutic Agents or Targets

As discussed above (see section “Testicular Cancer”), the involvement of IL6 in testis cancer and the suggested immuno-editing by neoplastic germ cells (61, 100, 106) renders this pro-inflammatory and pro-proliferative molecule a putative therapeutic target. Additional cytokine pathways involved with establishment and maintenance of the tumor microenvironment may be strong candidates for adjunct therapeutics, but much work remains to be done to reach this outcome.

In patients with acute mumps orchitis, *IFNα2B* with putative antiviral activity was administered in order to prevent or minimize testicular damage (164, 165). The therapeutic efficacy of this concept, however, was not convincing; in a follow-up study, testicular biopsies revealed total atrophy of seminiferous tubules in 38% of patients and partial atrophy in 16% (165).

As outlined above (see section “Inflammatory Conditions of the Testis: How Frequent and How Important Are They?”), TNF represents a hallmark of non-neoplastic testicular inflammation. Early experiments using the adoptive transfer of EAO in mice revealed that recipient pre-treatment with neutralizing antibody to TNF, but not neutralizing antibody to *IFNγ*, attenuated autoimmune orchitis (166). This concept, however, has not been revisited in a clinical setting for men suffering non-infectious (or post-infectious) orchitis, although available TNF inhibitors are routinely used for a wide range of chronic inflammatory diseases. To date, therapeutic attempts to improve chronic low-grade testicular inflammation have been limited to orally administered glucocorticosteroids, non-steroidal anti-inflammatory drugs such as cyclooxygenase inhibitors as well as mast cell stabilizers (110, 114).

Other therapeutic avenues will no doubt be identified, as research dedicated to the preservation of fertility in cancer patients are developed. Encouraging results have been obtained from the administration of granulocyte colony stimulating factor (G-CSF) to male rodents prior to the application of previously sterilizing doses of the chemotherapeutic agent, busulfan (167, 168). Important clues to its mode of action in this circumstance may emerge as we better understand how local immune cell functions influence spermatogenesis, as G-CSF is typically administered to support neutrophil function. Future developments may include application of anti-fibrotic treatments to prevent long-term testicular damage, emerging from investigations of agents that block TGFβ and activin signaling in the testis and epididymis during key stages of infection [e.g., Ref. (127, 169)].

### Potential Testicular Adverse Effects of Immunomodulatory Interventions

In recent years, a growing number of small molecules with immunomodulatory properties and biologics have become available and are routinely used in chronic inflammatory diseases as well as hematologic and other malignancies. Considering major improvements in survival rates and quality of life, potential long-term effects on male reproductive health are a matter of concern. Some examples are discussed below.

Inhibitors of TNF comprise monoclonal antibodies (e.g., infliximab) or fusion proteins (e.g., etanercept). Although effects of infliximab on spermatogenesis could be shown *in vitro*, no impairment of male fertility was observed clinically in patients with rheumatoid disease (170–173). Ustekinumab, a monoclonal IgG1 antibody that binds to the p40 subunit of IL12 and IL23, revealed no reproductive toxicity in male cynomolgus monkeys, whereas data from clinical studies are missing [reviewed in Ref. (174)]. For the upcoming range of antibodies targeting IL17A (e.g., in patients with psoriasis), neither pre-clinical nor clinical data concerning adverse effects on testicular and overall male reproductive function are available (175). Whereas the selective blockade of the IL1 receptor by anakinra does not seem to impair male fertility, semen quality even improved during treatment in patients suffering auto-inflammatory Muckle–Wells syndrome (176).

Animal studies with rituximab, a chimeric (mouse/human) anti-CD20 antibody achieving B cell depletion, showed no deleterious effects on reproductive organs (174). By contrast, administration of the anti-CTLA4 antibody ipilimumab, which interferes with T cell activation, showed a negative effect on testicular volume in pre-clinical experiments, whereas patients treated with this antibody are at risk of developing autoimmune endocrinopathies (i.e., hypophysitis) (177, 178).

Considering that alterations in the KITL/KIT system may cause disorders of the neuro-endocrine-immunological network, tyrosine kinases (mTOR inhibitors) such as imatinib should not be ignored. A small number of studies have examined the effect of imatinib administration on rodent spermatogenesis, with only one addressing fertility outcomes. Despite some measurable effects on testis growth and function when delivered to juveniles, healthy offspring were fathered by treated males (179, 180). Despite a lack of controlled clinical studies, case reports indicate that this drug can impair sperm quality, even resulting in azoospermia (181). On the other hand, imatinib decreased mast cell counts and tryptase release in patients with severe asthma (182).

## Concluding Remarks

Consolidation of knowledge regarding the roles of cytokines in development and maintenance of the testis is integral to making advances in diagnosis and treatment for male infertility, a condition which affects up to 1 in 20 couples. This review has provided examples of how information from basic research in non-mammalian species can address this need and highlighted the profound impact of immune cell function on normal and pathogenic functions in the testis. Future studies that detail the testis-specific function of specific cytokines and chemokines, and reveal their interactions with other cellular signaling pathways, will be essential to improve clinical management for patients with male infertility or with other conditions in which spermatogenesis may be impaired by the disease or its therapies.

## Author Contributions

All authors contributed to the text and edited the manuscript.

## Conflict of Interest Statement

The authors declare that the research was conducted in the absence of any commercial or financial relationships that could be construed as a potential conflict of interest.
